# The founder hypothesis: A basis for microbiota resistance, diversity in taxa carriage, and colonization resistance against pathogens

**DOI:** 10.1371/journal.ppat.1007563

**Published:** 2019-02-21

**Authors:** Yael Litvak, Andreas J. Bäumler

**Affiliations:** Department of Medical Microbiology and Immunology, School of Medicine, University of California at Davis, Davis, California, United States of America; Nanyang Technological University, SINGAPORE

## Introduction

Our skin and mucosal surfaces are colonized by diverse microbial communities, collectively known as the microbiota [1]. The microbiota provides benefits as microbial metabolites contribute to host nutrition and immune education, although the viability of germ-free animals conjectures that these two functions are not essential for life. However, environmental exposure makes germ-free animals prone to lethal infection, illustrating that the microbiota confers a third function that is often vital, namely, the ability to confer colonization resistance against pathogens [2].

Colonization resistance is an acquired trait, because the microbiota is assembled after birth by attaining maternal and environmental microbes [3]. To coexist, each species within the microbial community needs to be able to utilize a critical resource better than any other member of the microbiota, and the abundance of this growth-limiting resource determines the abundance of the species, a concept known as the nutrient-niche hypothesis [4]. The conceptual framework of the nutrient-niche hypothesis suggests that the neonate microbiota will mature until all discrete nutrient-niches have been filled with a suitable occupant, thereby reaching an equilibrium state [5]. Assuming the same anatomical location in different individuals exposes similar nutrient-niches, the nutrient-niche hypothesis further predicts that the metabolic pathways that enable each member within the microbial community to utilize its growth-limiting nutrient must be conserved between different individuals. Consistent with this prediction, metabolic pathways encoded by the microbiota are very similar between individuals [1]. However, carriage of microbial taxa varies greatly within a healthy population [1], an observation that is not explained by the nutrient-niche hypothesis and remains poorly understood.

### Priority effects generate variation in taxa carriage

Host genetic variation explains only a small fraction of taxonomic microbiota variation between individuals, whereas environmental influences dominate this trait [6]. An important environmental influence in the gastrointestinal tract is the diet, which determines the availability of a subset of growth-limiting nutrients, thereby adding or subtracting nutrient-niches [7, 8]. For example, microbiota-accessible carbohydrates found in dietary fiber determine the abundance of fiber-consuming saccharolytic bacteria in the gut microbiota, and prolonged dietary fiber starvation can lead to an irreversible extinction of species specialized in devouring this critical resource by eliminating their nutrient-niche [8]. Although diet can generate statistically significant changes in the taxonomic composition of the gut microbiota, these changes are small compared to the variation observed between individuals. Furthermore, diet does not provide a plausible explanation for the taxonomic diversity observed in microbial communities outside the gastrointestinal tract [1]. Instead, a critical factor generating taxonomic microbiota diversity between individuals is the order of species arrival and timing by which host surfaces are colonized early in life [9].

**Fig 1:**
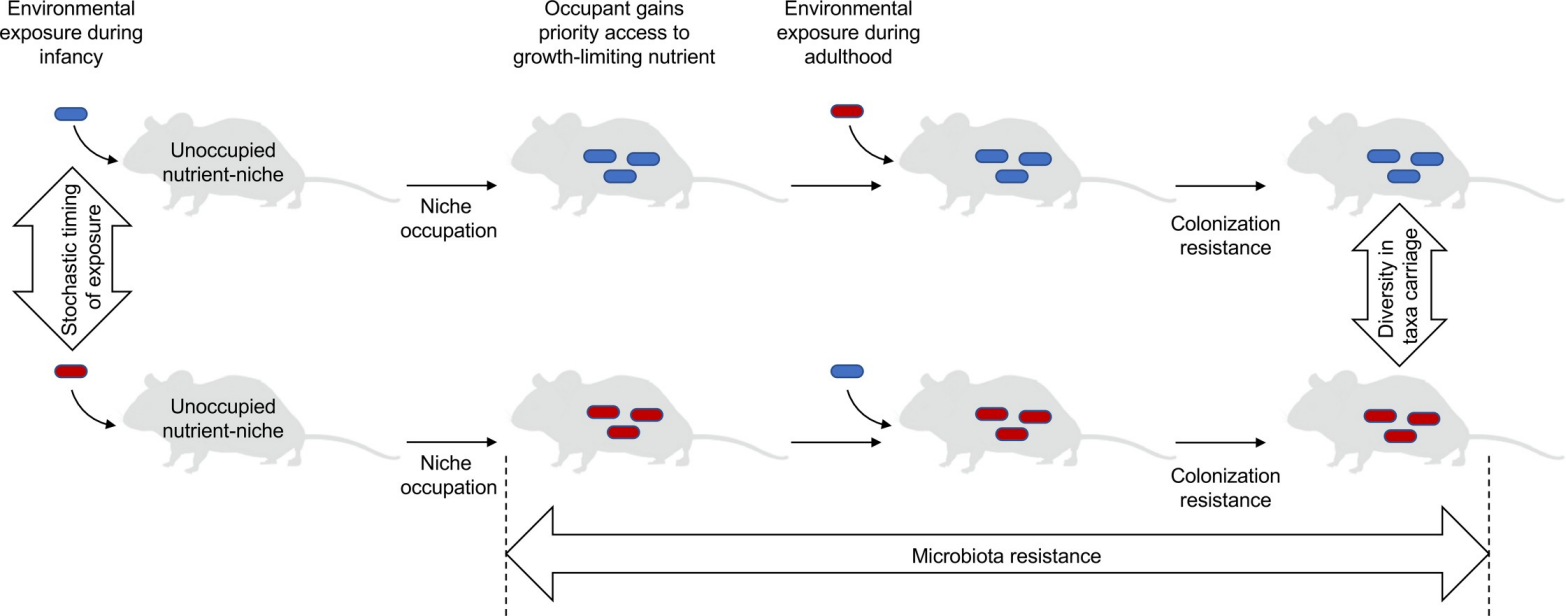
The founder hypothesis. The principles of the founder hypothesis are shown schematically for a single nutrient-niche. Stochastic effects governing microbial exposure during infancy determine which microbial species (red or blue rods) establishes residency in the nutrient-niche, thereby generating diversity in taxa carriage between individuals. The founding occupant gains priority access to the growth-limiting resource that defines its nutrient-niche. These priority effects enable the occupant to confer colonization resistance against environmental exposure to microorganisms that are suitable contenders for the same nutrient-niche. The resulting resistance to stress imposed through environmental exposure to microorganisms produces microbiota resistance.

The colonization order influences both the outcome of microbial community assembly and the ecological success of individual microbes [3, 9]. These priority effects are preserved in mice lacking adaptive immunity, suggesting that acquired host responses are not a major source of taxonomic diversity in the microbiota composition [9]. Priority effects are mediated through niche preemption or niche modification and can involve the genetic adaptation of microbes to a niche [9, 12], but the underlying mechanisms are incompletely understood. Mechanistic insights into this “first come, first serve” phenomenon suggest that the microbe that initially occupies a nutrient-niche in a neonate gains priority access to the growth-limiting nutrient that defines its nutrient-niche [10]. A growth-limiting resource that determines the abundance of facultative anaerobic *Enterobacteriaceae* (phylum Proteobacteria) within the microbiota of the large intestine is the availability of respiratory electron acceptors, such as oxygen [11]. *Escherichia coli* (family Enterobacteriaceae) has access to oxygen in the ceca of neonate chicks when it is inoculated one day prior to challenge with *Salmonella enterica* (family Enterobacteriaceae) but not when neonate chicks receive both species at the same time [10], suggesting that order and timing of gut colonization determine whether growth-limiting resources are accessible to a microbe. Henceforth we will refer to the concept that the founding occupant gains priority access to the growth-limiting resource that defines its nutrient-niche as the “founder hypothesis.” The founder hypothesis suggests that stochastic effects that govern the initial exposure of neonates to microbes that become founding occupants of each nutrient-niche are a prominent source of taxonomic variation in the microbiota composition between individuals (Fig 1) [3].

### Priority effects generate microbiota resistance

The composition and function of mature microbial communities within an individual host is relatively stable against perturbations, a phenomenon known as microbiota resistance [5]. For instance, microbiota profiling of healthy human adults suggests that the vast majority of strains in the fecal microbiota of an individual are residents for years [13]. The stability of the microbiota composition within an individual over time is surprising, given our daily exposure to a steady stream of microbes that challenge the microbiota using their antibacterial weaponry, such as colicins, contact-dependent growth inhibition systems, or type VI secretion systems [14]. The compositional stability of the microbiota predicts that the resident microbes must be able to withstand the stress imposed by environmental exposure to new microbial species, but the mechanistic underpinnings remain unknown. An appealing aspect of the founder hypothesis is the prediction that it is difficult for newly arriving microbes to replace those already present, because priority access to a growth-limiting resource provides the founding occupant with a competitive advantage. As a result of the selective advantage conferred by priority effects, the microbiota composition remains resilient to change, despite the continuous stress imposed by exposure to antibacterial weaponry of microbial intruders (Fig 1). Therefore, the founder hypothesis provides a conceptual framework to explain the compositional stability and resistance of the gut microbiota [13].

A correlate of the nutrient-niche hypothesis is that an antibiotic-mediated extinction of strains from the gut microbiota should be followed by an acquisition of new microbes, each encoding a metabolic pathway needed to fill one of the nutrient-niches vacated by the elimination of an antibiotic-sensitive microbe. The founder hypothesis predicts that these newly acquired microbes will be steadily maintained subsequently through priority effects, thereby resulting in a permanent shift in the microbiota composition. Consistent with these projections, daily gut microbiota profiling over the course of one year shows that the gut microbiota composition in one individual was stable until an episode of gastroenteritis required antibiotic therapy. Antibiotic treatment was associated with a permanent decline of most gut bacterial taxa, which were replaced subsequently with new species that were genetically similar and were afterward maintained steadily for the remainder of the study [15]. Therefore, the occupation of all available nutrient-niches early in life leads to an equilibrium state that remains relatively stable during adulthood [5]. However, perturbation with antibiotics can lead to a permanent replacement of antibiotic-sensitive microbes by new founders that are acquired randomly from the environment after the cessation of treatment. Once all nutrient-niches are occupied again, the microbiota returns to a stable equilibrium state, but the result is a permanent shift in the microbiota composition [15].

### Priority effects contribute to colonization resistance

A mature microbiota confers colonization resistance, because each of its members occupies one of the available nutrient-niches (nutrient-niche hypothesis) and holds a competitive advantage over newly arriving microbes through priority effects (founder hypothesis). As a result, it is difficult for newly arriving microbes to establish permanent residency because the best seats in the house are already taken (Fig 1), which explains why ingestion of probiotics has only a transient impact on the microbial community structure in healthy individuals [12, 16]. One way to overcome colonization resistance is to clear a nutrient-niche by removing its occupant with an antibiotic and filling the resulting void with a suitable microorganism. This mechanism clarifies why antibiotic therapy can prolong fecal shedding of probiotics in humans [12, 17] and predispose patients to infection with enteric pathogens [2, 18].

An alternate strategy for overcoming colonization resistance is to generate an additional nutrient-niche that allows a new microbe to be added to the microbiota [7]. In the gastrointestinal tract, new nutrient-niches can be generated by the addition of a complex carbohydrate, such as a prebiotic, to the diet [19]. Synbiotics are dietary supplements that combine a prebiotic with a probiotic with the goal to create a new nutrient-niche with the complex carbohydrate to improve the viability of a microorganism consuming this resource [7]. A similar strategy is used by Mother Nature to generate a nutrient-niche for *Bifidobacterium infantis* (phylum Actinobacteria) in breast-fed infants, as human milk oligosaccharides are a growth-limiting nutrient for this microbe [20]. Weaning eliminates the nutrient-niche for *B*. *infantis*, which results in a loss of the microbe from the human gut microbiota.

Mucosal pathogens can establish residency in the microbiota of apparently healthy individuals using a strategy that involves neither antibiotics nor dietary supplements. To solve the challenge that all available nutrient-niches are occupied and the occupants are difficult to remove, mucosal pathogens use their virulence factors to create a new nutrient-niche for themselves by eliciting host responses that alter the environment at the mucosal surface [11]. Upon entry, mucosal pathogens initially find themselves in a precarious situation, because prior to the development of host responses, microbiota-mediated colonization resistance blocks their access to nutrient-niches, thereby curbing pathogen growth. The resulting decline in pathogen numbers can lead to an extinction, an outcome that becomes more likely when the host is exposed to a low infectious dose. However, when the infectious dose exceeds a critical threshold needed for eliciting host responses that create a new nutrient-niche, the pathogen can establish residency and expand [11]. The host responses generated during this process make us sick, which is one of the reasons why we refer to microorganisms using this strategy as pathogens, but the goal from the pathogen’s point of view is simply to gain access to a growth-limiting resource [21].

### Future directions

An unexplained diversity in taxa carriage across individuals and the poorly understood phenomenon of microbiota resistance support the idea that a theoretical framework for hypothesis-driven research is largely still lacking in microbiome studies [22]. Here, we make the case that the nutrient-niche and founder hypotheses can provide a conceptual framework for hypothesis-driven research on central questions in microbiota research. The looming implications of the founder hypothesis stand in contrast to the relatively small amount of information available on the mechanistic basis for priority effects. We argue that investment in research on the mechanistic underpinnings of priority effects will greatly enhance our understanding of how host-associated microbial communities function during health and disease and usher in an era of mechanistic microbiota research.
